# Atypical Fibroxanthoma Presenting as a Rapidly Enlarging Tender Papule: Case Report

**DOI:** 10.2196/84550

**Published:** 2026-10-30

**Authors:** Elizabeth Tchernogorova, Nicole Skalka, Mary Tidwell, Binh Minh Nguyen, Julien Lanoue, Robin Burger, McKenna Abercrombie

**Affiliations:** 1 Rocky Vista University, Ivins, UT, United States; 2 Rocky Vista University, 8401 S Chambers Rd, Englewood, CO, United States, 1 3033732008; 3 Advanced Dermatology and Cosmetic Surgery, Brandon, FL, United States; 4 Advanced Dermatology and Cosmetic Surgery, Delray Beach, FL, United States

**Keywords:** atypical fibroxanthoma, mesenchymal neoplasm, sun-exposed skin, Mohs micrographic surgery, immunohistochemistry, skin cancer

## Abstract

**Background:**

Atypical fibroxanthoma (AFX) is a rare dermal mesenchymal neoplasm that is most commonly identified on sun-exposed areas of the head and neck of older adults. Presentation on the dorsal hand is uncommon.

**Objective:**

This report presents a rare case of AFX, its clinical presentation, histopathology, and treatment, and discusses the use of immunohistochemistry as a method in diagnosing AFX.

**Methods:**

We evaluated a 67-year-old woman with a history of basal cell and squamous cell carcinomas who presented with a rapidly enlarging, tender papule on the dorsal aspect of her left hand. Prior topical 5-fluorouracil therapy was ineffective. Histopathologic examination demonstrated findings favoring AFX, and treatment was initiated. A literature review was performed to contextualize this case among previously reported instances of AFX.

**Results:**

Clinical examination revealed a 1.2-cm, dome-shaped, erythematous papule. Histopathology demonstrated a dermal-based pleomorphic spindle cell neoplasm with brisk mitotic activity. Immunohistochemistry showed positivity for CD68 and CD10, with negative staining for SOX10, PRAME, pankeratin, p40, SMA, desmin, and CD34. The morphologic and immunohistochemical findings favored AFX. The tumor was completely excised via Mohs micrographic surgery (MMS) in a single stage with negative margins. No adjuvant therapy was required.

**Conclusions:**

AFX remains a diagnosis of exclusion requiring comprehensive immunohistochemistry to distinguish it from morphologic mimics with aggressive biologic behavior. This case highlights that MMS is the preferred treatment for AFX, offering the lowest recurrence rates with maximal tissue conservation.

## History

A 67-year-old woman presented with a rapidly enlarging, tender papule on the dorsal aspect of her left hand. The lesion had evolved over several weeks, with no preceding trauma or known immunosuppression. The patient reported significant lifetime sun exposure and a history of non-melanoma skin cancer.

## Ethical Considerations

Written informed consent was obtained from the patient pertaining to the use of their clinical photographs and medical information to be published online and with the understanding that this information may be publicly available and discoverable via search engines. Patient consent forms were provided to the journal.

## Examination

Physical examination revealed a solitary, erythematous, dome-shaped papule measuring approximately 1.2 cm in diameter (Figure 1). The lesion was firm, nonulcerated, and tender to palpation. No regional lymphadenopathy was detected. The remainder of the cutaneous exam was unremarkable.

**Figure 1.**
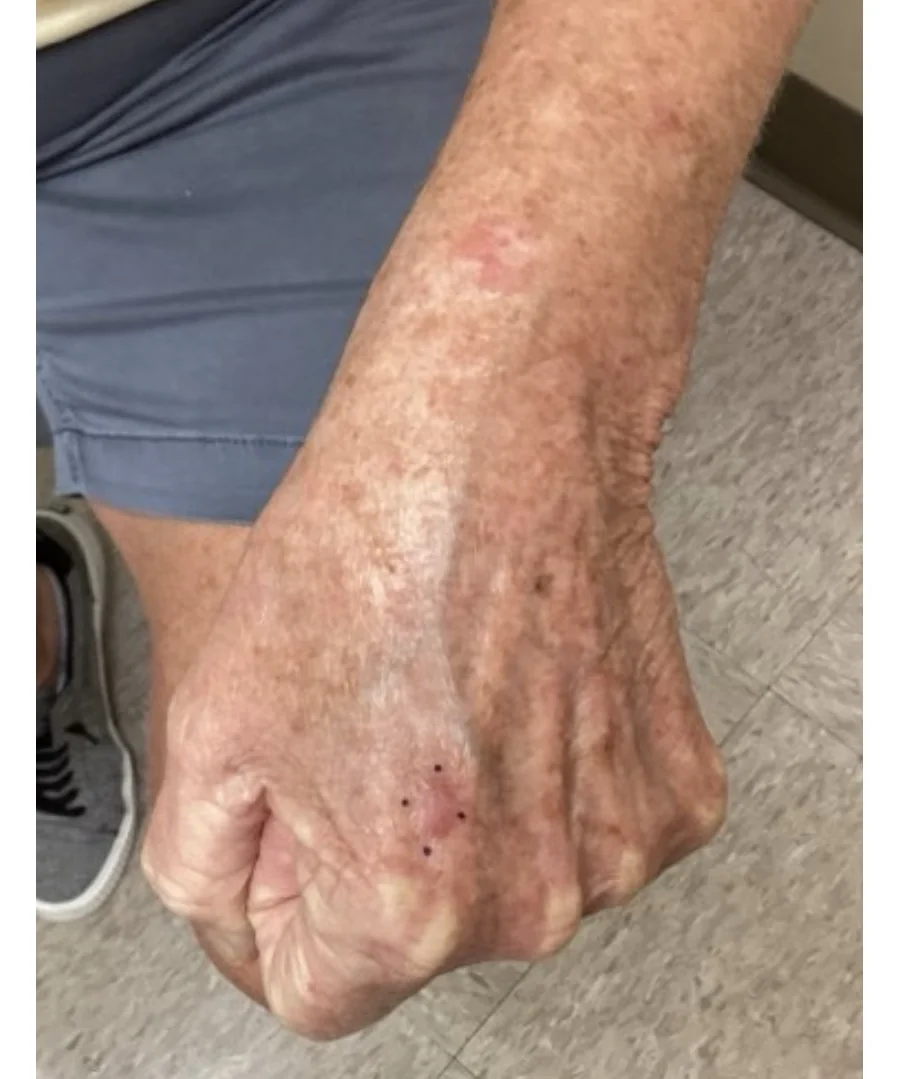
Papule on the dorsal aspect of patient’s left hand.

## Histopathology

A shave biopsy demonstrated a dermal-based neoplasm composed of pleomorphic spindle and epithelioid cells with hyperchromatic nuclei and abundant cytoplasm (Figure 2, Figure 3). Histopathologic findings favored atypical fibroxanthoma (AFX). Given the superficial nature of the biopsy, however, the possibility that the lesion represented the superficial portion of a pleomorphic dermal sarcoma cannot be entirely excluded. Excision of any residual lesion was recommended for complete histologic evaluation. The lesion stained positive for CD68 and CD10 and negative for SOX10, PRAME, pankeratin, p40, SMA, desmin, and CD34. In the appropriate morphologic context, the immunohistochemical findings supported the diagnosis of atypical fibroxanthoma by helping to exclude epithelial, melanocytic, and smooth muscle differentiation.

**Figure 2.**
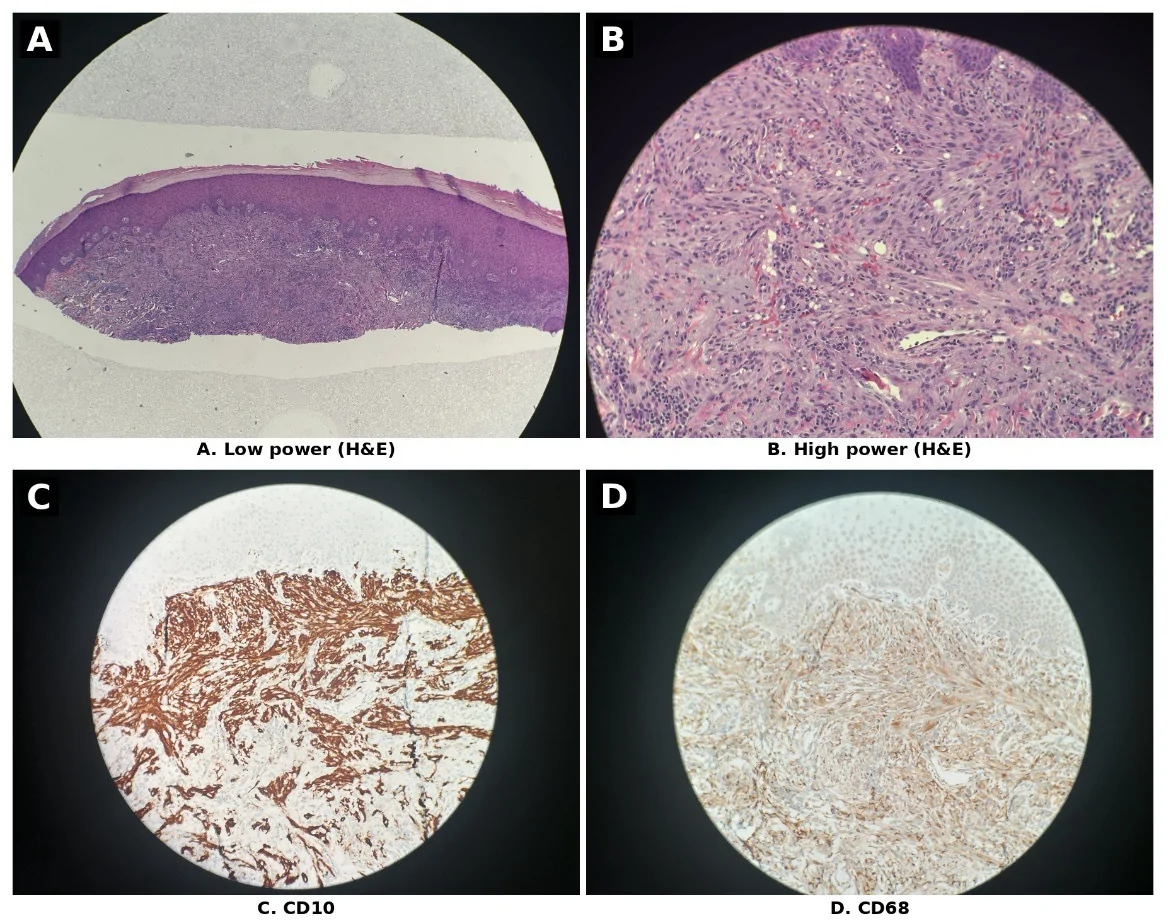
Histopathologic and immunohistochemical findings from the shave biopsy. (A) Low-power hematoxylin and eosin (H&E) stain demonstrates a dermal-based spindle cell proliferation (x4). (B) High-power H&E demonstrates a proliferation of atypical pleomorphic spindle cells (x20). (C) Lesional cells demonstrate strong immunoreactivity for CD10. (D) Lesional cells demonstrate immunoreactivity for CD68.

**Figure 3.**
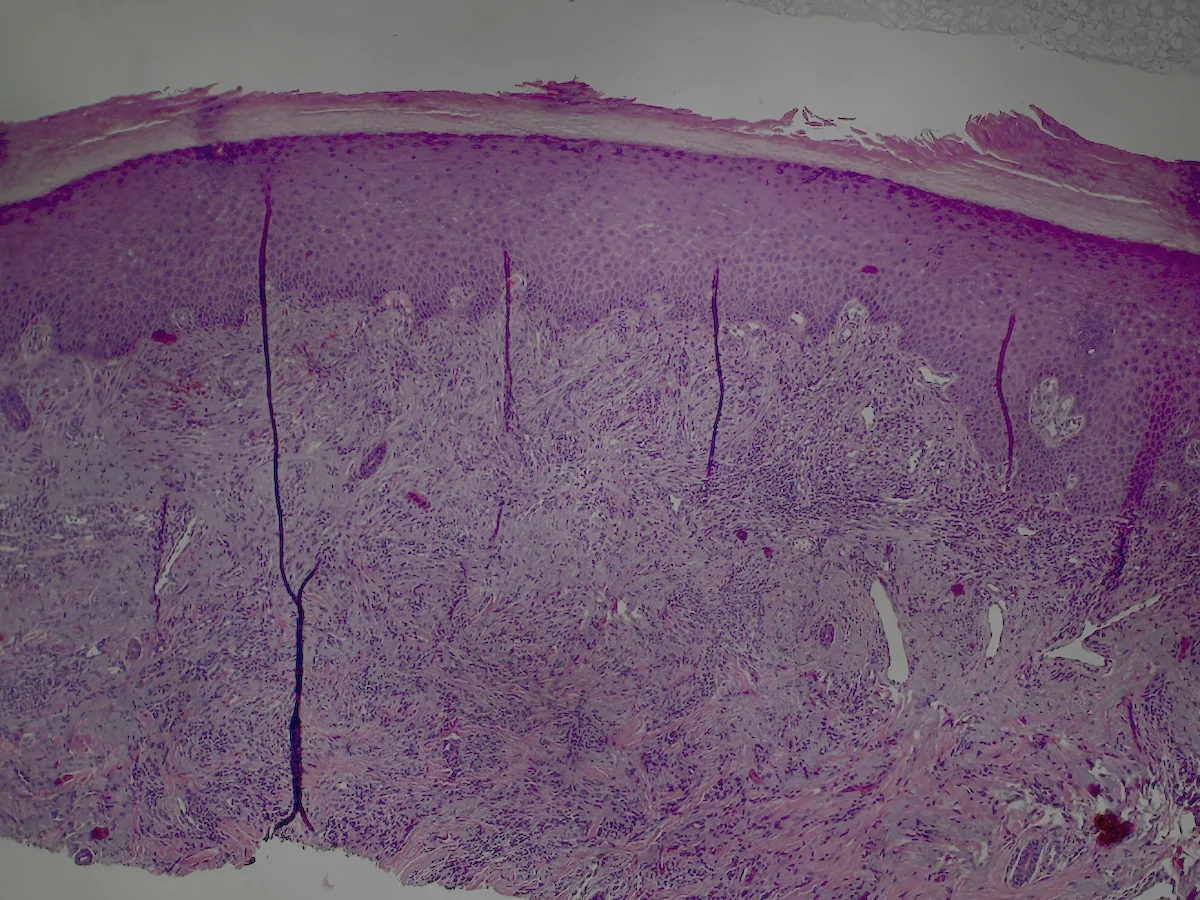
Histopathology of the lesion.

## Course and Therapy

The patient was referred to Mohs micrographic surgery, a treatment associated with a high cure rate (Figures 4–6), where the tumor was cleared in a single stage with histologically negative margins. No adjuvant therapy was necessary. In the absence of residual tumor for further evaluation, and given the morphologic and immunohistochemical findings of the original biopsy, the lesion was regarded as atypical fibroxanthoma.

**Figure 4.**
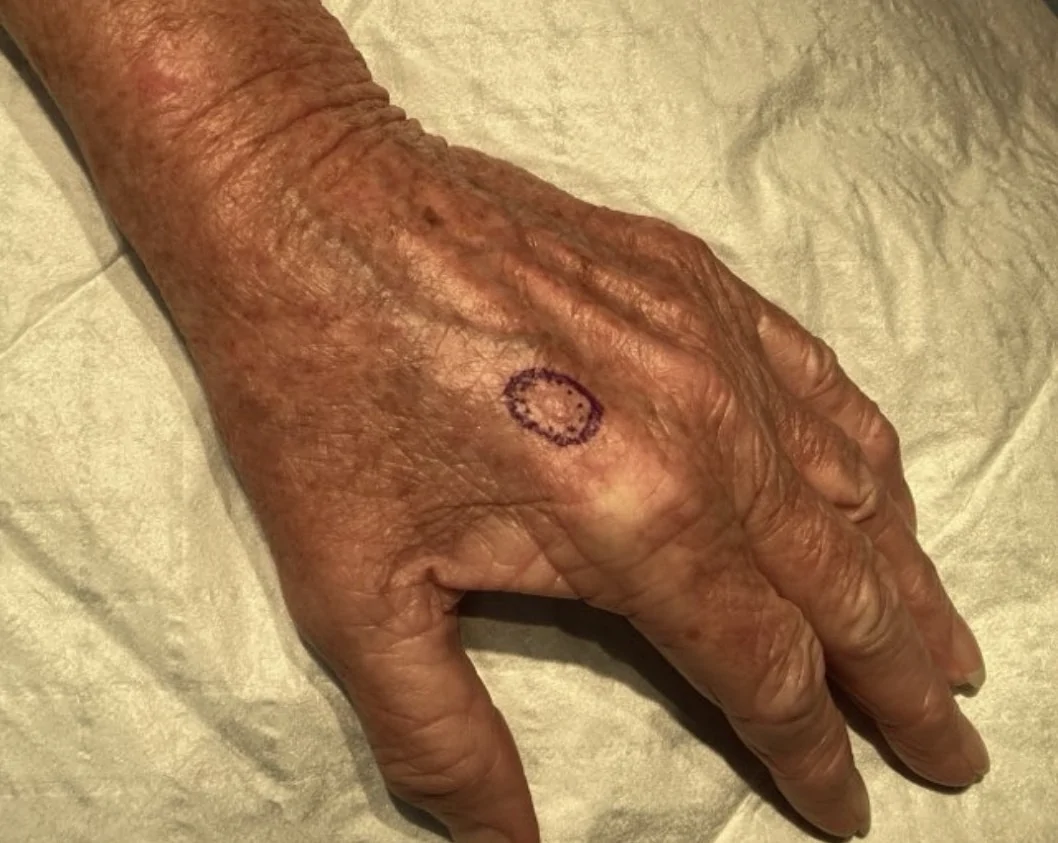
Pre-operative marking of the papule margins.

**Figure 5.**
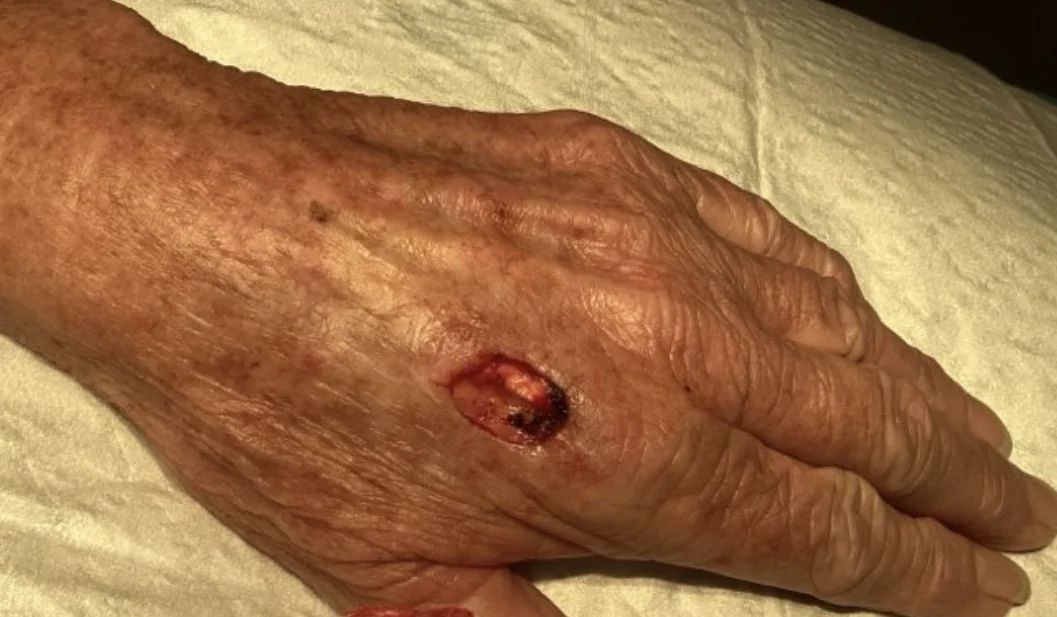
Papule site post operation showing negative margins.

**Figure 6.**
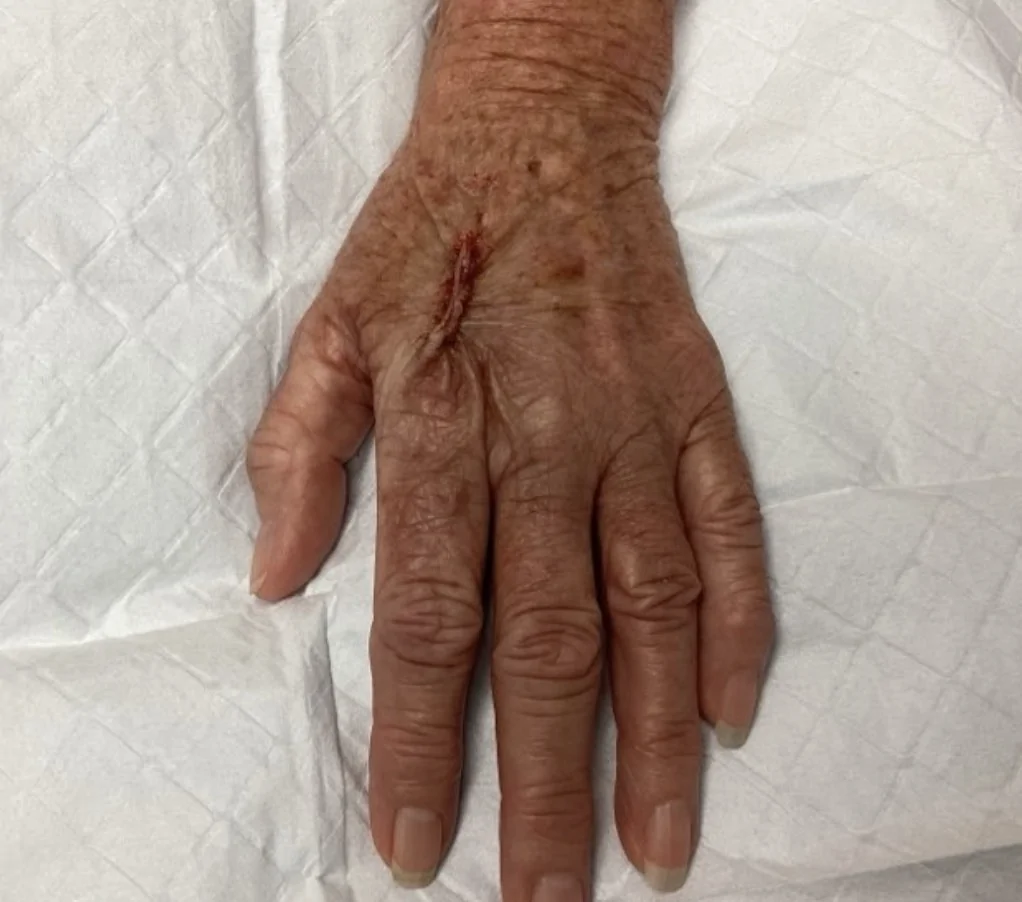
Repair closure after completed Mohs micrographic surgery.

## Discussion

AFX is a rare mesenchymal neoplasm that has been reported with increasing frequency over the past decade [1,2]. It is most commonly identified on sun-exposed areas of the head and neck of older adults, with a mean age of 75.8 years [1,3]. Approximately 89% of lesions arise in the head and neck; presentation on the dorsal hand is uncommon [4]. The diagnosis of AFX is often one of exclusion, as it shares overlapping morphological features with other neoplasms that develop as a result of chronic sun exposure [1]. Ultraviolet (UV) exposure is a known risk factor for the development of AFX due to its ability to induce mutations in p53, a tumor suppressor protein [5,6]. Histologically, AFX can resemble undifferentiated pleomorphic sarcoma [1,3,5,7,8]. These tumors are typically well-circumscribed but not encapsulated, showing an expansile, non-infiltrative growth pattern and are usually confined to the superficial dermis. In some cases, they may extend deeper into the reticular dermis. A diagnostically useful feature is the lack of a dermal “grenz zone,” a band of normal papillary dermis spared by the lesion [1]. The apparent rise in AFX diagnoses may in part be attributed to increased use of immunohistochemical panels, which aid in distinguishing AFX from histologic mimickers [2]. Commonly used markers include pan-cytokeratin, S100, desmin, CD34, CD10, procollagen-1, CD99, CD14, and CD68 [2,9]. Early biopsy is critical for accurate diagnosis and prompt surgical intervention.

When considering a differential diagnosis, the primary entities of concern include spindle cell squamous cell carcinoma, malignant melanoma, leiomyosarcoma, undifferentiated pleomorphic sarcoma, and dermatofibrosarcoma protuberans [5,6]. As noted, both clinical presentation and immunohistochemical staining are critical for distinguishing these tumors [2]. Standard treatment of AFX is complete surgical excision with margin control, most commonly achieved through Mohs micrographic surgery. Surgical treatment has shown lower local recurrence rates, as well as optimal cosmetic outcomes. A wide local excision with a margin of at least 1‐2 cm can be used if MMS is not feasible. Margin control is critical when using a wide local excision, and incomplete excision can result in recurrence. Given AFX’s low metastatic potential, adjuvant therapy is generally not necessary. In general, prognosis is excellent because of low metastatic potential and adequate excision margins [7].

In this report, we discuss a unique case of AFX presenting on the dorsal aspect of the hand. Typically, AFX occurs in sun-exposed areas of the head and neck, such as the cheeks, nose, forehead, and scalp [10]. Instances of AFX appearing outside the head and neck have rarely been documented. Despite this patient’s atypical location, the patient achieved a successful outcome with single-stage MMS. In summary, this case underscores the importance of considering AFX in less common sites and emphasizes that prompt recognition and treatment with MMS are crucial to ensure the best possible outcome.

## Abbreviations

AFX: atypical fibroxanthoma
MMS: Mohs micrographic surgery
